# Therapeutic Biomaterial Approaches to Alleviate Chronic Limb Threatening Ischemia

**DOI:** 10.1002/advs.202003119

**Published:** 2021-02-08

**Authors:** Grazia Marsico, Sergio Martin‐Saldaña, Abhay Pandit

**Affiliations:** ^1^ CÚRAM SFI Research Centre for Medical Devices National University of Ireland Galway Ireland

**Keywords:** biomaterials, cells, chronic limb threatening ischemia, growth factors, nucleic acids

## Abstract

Chronic limb threatening ischemia (CLTI) is a severe condition defined by the blockage of arteries in the lower extremities that leads to the degeneration of blood vessels and is characterized by the formation of non‐healing ulcers and necrosis. The gold standard therapies such as bypass and endovascular surgery aim at the removal of the blockage. These therapies are not suitable for the so‐called “no option patients” which present multiple artery occlusions with a likelihood of significant limb amputation. Therefore, CLTI represents a significant clinical challenge, and the efforts of developing new treatments have been focused on stimulating angiogenesis in the ischemic muscle. The delivery of pro‐angiogenic nucleic acid, protein, and stem cell‐based interventions have limited efficacy due to their short survival. Engineered biomaterials have emerged as a promising method to improve the effectiveness of these latter strategies. Several synthetic and natural biomaterials are tested in different formulations aiming to incorporate nucleic acid, proteins, stem cells, macrophages, or endothelial cells in supportive matrices. In this review, an overview of the biomaterials used alone and in combination with growth factors, nucleic acid, and cells in preclinical models is provided and their potential to induce revascularization and regeneration for CLTI applications is discussed.

## Graphical abstract

Herein, the most recent advances of biomaterial design applied to chronic limb threatening ischemia (CLTI) are reviewed. Several biomaterial‐based formulations are used alone and in combination with growth factors, nucleic acid, exosomes, and cells in preclinical models. Their potential to induce revascularization and future directions to overcome actual limitations when treating CLTI are critically discussed.

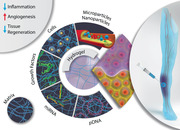

## 1 Introduction

Chronic limb threatening ischemia (CLTI) is a peripheral vascular disease that occurs with the occlusion of arteries of the lower extremities, mainly due to the formation of the atherosclerotic plaque.^[^ ^1^ ^]^ CLTI has an incidence of 500–1000 new cases per million individuals every year in Western society.^[^ ^2^ ^]^ Being the advanced stage of peripheral artery disease, it is an increasing public health concern due to the rising in global prevalence in cardiovascular risk factors. CLTI also imposes a substantial burden on patients, healthcare providers, and resources.^[^ ^3^ ^]^ Mortality rates due to CLTI and related prognosis could exceed 50% within five years of diagnosis. Albeit, the so‐called “no‐option CLTI patients,” that are not eligible for revascularization, present a mortality range from 20% to 40% during the first year.^[^ ^3^ ^]^

The pathophysiology of CLTI, caused by the reduction of blood supply, leads to a complex scenario involving hypoxia, oxidative stress, macro‐ and microvascular dysfunctions, inflammation, and muscle fiber necrosis.^[^ ^3^ ^]^ The symptoms of CLTI patients are pain at rest, intermittent claudication in the early stages, non‐healing ulcers, and gangrene at the later stages. In addition to pain relief and wound healing, CLTI treatments aim to control the symptoms and the revascularization of the lower limb. Functional limb recovery and survival rate improvement, as well as patient quality of life, are the critical goals of CLTI clinical management.^[^ ^4^ ^]^ Revascularization strategies, currently in use, involve surgical intervention through endovascular techniques or bypass that aim at removing or bypassing the occlusion.^[^ ^3^ ^]^

**Figure 1:**
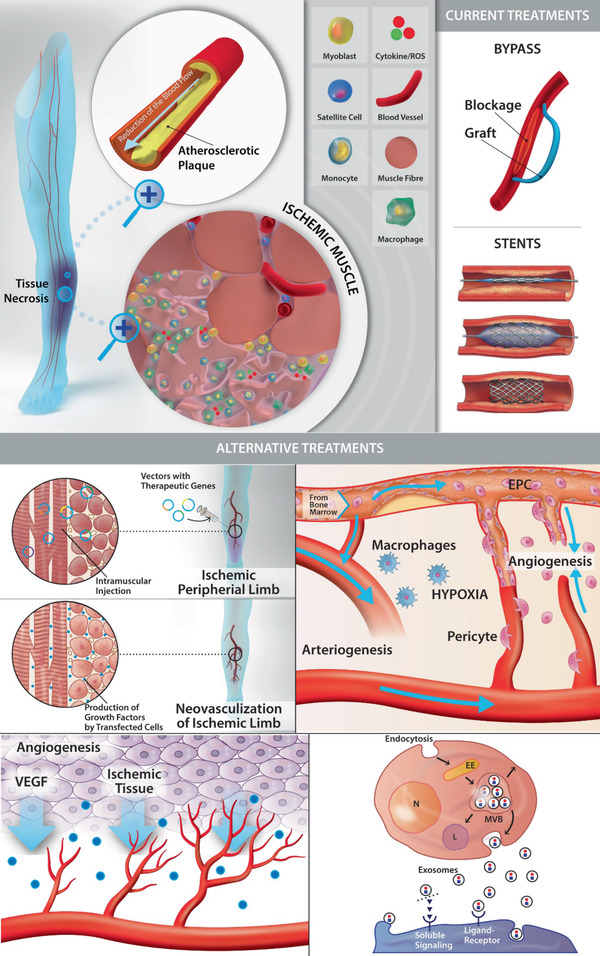
Schematic representation of the pathological scenario of CLTI, and the current and alternative treatments in clinic. CLTI arises when occlusion of arteries of the lower limb occurs, most probably due to the formation of the atherosclerotic plaque in the arterial wall. The resulting reduction in blood flow and the consequent lack of oxygen and nutrients causes the necrosis of skeletal muscle. The damaged muscle fibers present inflammatory cell infiltration at the necrotic segments. The damage triggers the differentiation of the satellite cells, present in the intact segments, into myoblasts that starts a responsive regeneration process. Current treatments are based on revascularization strategies involving surgical intervention through bypass or endovascular techniques (stents and balloons). As alternative treatments, from left to right, the use of nucleic acid, cell based therapies, growth factors and exosomes have been blossomed in the last decade with promising results in clinical trial. Abbreviations: EPC, endothelial precursor cell; VEGF, vascular endothelial growth factor; N, nucleus; EE, early endosome; L, lysosome; MVB, multi‐vesicular bodies.

Both endovascular procedures and bypass surgeries are indicated if the patient is affected by a significant localized occlusion. When patients present salvageable limbs, the gold standard approach is revascularization to improve and/or recover distal perfusion or both. As described above, revascularization offers a range of surgical options, such as open surgery, including arterial bypass and endarterectomy. Besides, endovascular treatment may be more effective and alternative methods, such as angioplasty or stent use, may be implemented in this situation. However, “no option patients” are those with multiple co‐morbidities and occlusions in smaller arteries. Consequently, these patients are not eligible for revascularization and will undergo primary limb amputation.^[^ ^5^ ^]^ Therefore, alternative treatments to address this clinical need are urgently required (**Figure** **1**).

The high complexity of CLTI's clinical scenario has led to the development of a plethora of different approaches. Over the past decade, growth factors/cytokines have been among the main protagonists of research efforts and clinical trials for CLTI. Despite encouraging results, the clinical efficacy is limited by the short half‐life in the pathological environment.^[^ ^3^ ^]^ The delivery of pro‐angiogenic growth factors, nucleic acids, and stem or vascular cells^[^ ^6^ ^]^ and cell‐free therapies have been the focus of the emerging therapeutic strategies.^[^ ^6^, ^7^ ^]^ Many of these non‐traditional strategies have shown their promising effects in pre‐clinical and, in some cases, clinical trials promoting the formation of new blood vessels.^[^ ^8^ ^]^ However, despite the beneficial outcomes, each of these approaches continue to present several translational challenges and limitations.^[^ ^9^ ^]^

The efficacy of growth factors or recombinant proteins in stimulating angiogenesis is limited by their short half‐lives.^[^ ^10^ ^]^ The administration of growth factors also showed adverse effects in preclinical models.^[^ ^11^ ^]^ Specifically, the administration of fibroblast growth factor‐2 (FGF‐2) caused hypotension,^[^ ^12^ ^]^ while vascular endothelial growth factor (VEGF) caused the formation of leaky blood vessels and tissue edema.^[^ ^11^ ^]^ In fact, despite improved hemodynamic measures in CLTI, FGF, hepatocyte growth factor (HGF) or VEGF did not prevent death or severe limb amputation or improve walking ability.^[^ ^13^ ^]^ Furthermore, excessive doses of the growth factor can generate some adverse effects. Indeed, the administration of FGF‐2 in high dose caused hypotension while VEGF resulted in the formation of leaky blood vessels and edema. The combined delivery of FGF‐2 and VEGF, instead, was associated with a rise of diabetic retinopathy and nephropathy.^[^ ^14^ ^]^

Moreover, co‐morbidities such as diabetes and hyperlipidemia can reduce the efficacy of growth factors.^[^ ^15^ ^]^ The use of pro‐angiogenic cytokines and growth factors have failed mainly due to the lack of proper preclinical models. This inadequacy of the models had led to small clinical trials with encouraging results that rapidly showed unsuccessful results in more extensive randomized clinical trials due to the lack of information regarding dosage, half‐life, and successful delivery of angiogenic cytokines. Mimicking angiogenesis is one of the biggest clinical challenges that is impossible to achieve with the administration of a single molecule due to its high complexity. All the cytokine growth factor based clinical trials have been failed in their primary ends. For that reason, there is no FDA‐approved (approved by the Food and Drug Administration) treatment for CLTI based on growth factors or cytokines.

Nucleic acid delivery could potentially overcome the main drawback of the protein‐based treatments, which deal with an impaired downstream in angiogenesis pathways.^[^ ^16^ ^]^ Nevertheless, its limitations include inadequate in vivo transfection efficacy and, on the other hand, the risk of developing tumor‐related aberrations due to persistent overexpression of growth factors.^[^ ^17^ ^]^ HGF angiogenic gene therapy is a case of particular interest since it showed promissory results in phase II and III of clinical trials.^[^ ^18^ ^]^ Powell et al. reported that HGF‐gene therapy was able to increase transcutaneous oxygen pressure when compared with placebo‐treated patients of CLTI.^[^ ^19^ ^]^ However, the trial (funded by AnGes USA Inc) stopped prematurely without revealing the final result of the trial. Further clinical trials in this direction are needed to understand the performance of nucleic acid delivery in clinical scenario.

The use of cell‐based therapeutics is an emergent strategy that has promise in the treatment of ischemic diseases. Cell therapies using mesenchymal stem cells,^[^ ^6^ ^]^ marrow‐derived CD34+ cells,^[^ ^20^ ^]^ mononuclear cells, and endothelial cells have been successful in several preclinical trials. Furthermore, the use of autologous stem cells is drawing attention due to their immuno‐privileged safety profile. Cell‐based therapies have been successful in reducing the mortality (less than 15%)^[^ ^21^ ^]^ but not in reducing the amputation rates among CLTI patients.^[^ ^22^ ^]^ Despite the promising results achieved in small clinical trials, larger clinical trials have failed to confirm the initial excitement surrounding cell‐based therapies. However, the fact that CLTI treatment is still a clinical need, there is evidence that has demonstrated significant improvement in patients, supports continued research has been fuelling the implementation of cell‐based therapies.^[^ ^23^ ^]^ As an example, the use of autologous human CD34+ stem cells had reduced amputation rate by improving perfusion and functionality in ischemic tissues. However, recent meta‐analysis of the clinical trials using CD34+ mononuclear cell therapy concluded that the reported results might not support its therapeutic benefit.^[^ ^24^ ^]^ As in the case of cytokine and growth factors, one of the most significant limitations of cell therapies is the lack of suitability of most of the preclinical models that do mimic the real pathophysiology of CLTI.

Several studies have shown that stem cells exert their therapeutic effect mainly through a paracrine mechanism.^[^ ^25^ ^]^ In this sense, exosomes have emerged as a critical paracrine tool of stem cells to reprogram injured cells in ischemic events.^[^ ^26^ ^]^ Exosomes are nano‐sized structures that actively deliver proteins or RNA through different biological interactions with other cells. The feasibility of improving natural exosomes or engineer synthetic ones and their high tunability allows the delivery of specific clues in order to promote angiogenesis.^[^ ^27^ ^]^ Nevertheless, exosomes systemic injection has resulted in the accumulation at undesired organs such as the liver or lung. Another drawback is related to a rapid clearance from blood in the first hours after the injection. A similar scenario has been reported when topically administered, with exosomes suffering a similar fate.

As can be noticed, there are an immense plethora of tools for CLTI medical intervention. Albeit the fact that most of them mitigate some of the main symptoms associated with CLTI, the ischemic damage is not fully addressed, leading to adverse prognosis. Biomaterial systems have emerged as an alternative strategy to overcome the limitations of the therapies mentioned above. Indeed, in the last 20 years, engineered biomaterials have been used as drug delivery systems and as a supportive matrix for prolonging cell survival in several preclinical applications.^[^ ^28^ ^]^ Mainly, various biomaterial systems have been used both alone and in combination with growth factors, genes, gasotransmitters, exosomes, and natural or synthetic cells in several CLTI preclinical models where they have been shown to induce tissue regeneration and angiogenesis.^[^ ^15^, ^29^ ^]^ However, despite promising preclinical results, the therapeutic efficacy of biomaterial‐based for CLTI has yet to be validated at the clinical level. Indeed, only a gelatin hydrogel/microspheres system with FGF has been shown to improve clinical outcomes in patients.^[^ ^30^ ^]^ This review aims to provide a critical overview of the current clinical scenario, the recent advances in the combinatorial and non‐biomaterial‐based therapies, as well as a discussion of the future direction in relation to materials development and optimization to overcome actual limitations when treating CLTI.

## 2 Biomaterials Application for CLTI

When engineering biomaterials to treat ischemic diseases is pivotal to promote blood reperfusion by encouraging the formation of new blood vessels.^[^ ^29a^ ^]^ As such, these biomaterials must modulate cell fate by promoting cell adhesion and migration,^[^ ^31^ ^]^ and provide mechanical support that triggers proliferation signals via mechanotransduction.^[^ ^32^ ^]^

**Figure 2:**
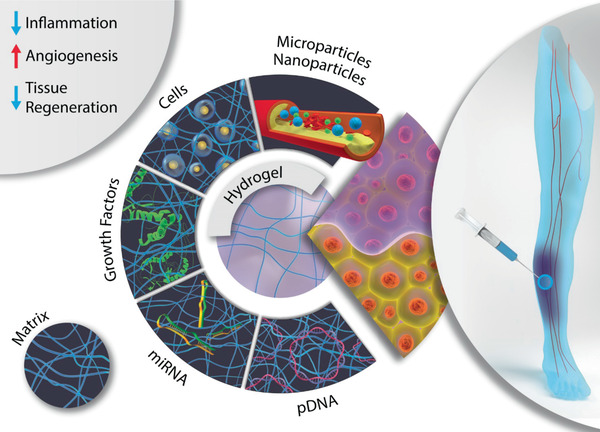
Schematic representation of the therapeutic strategies involving biomaterials. Preclinical trials have been lately focused on biomaterial alone therapies or biomaterials loaded with growth factors, nucleic acids (pDNA or micro ribonucleic acids (miRNA)), and cells (endothelial cells, macrophages, stem cells, and therapeutics) as potential treatment options for CLTI.

Several biomaterial‐based approaches natural and synthetic, protein or polysaccharide‐based, with different architectures have been investigated for CLTI applications (**Figure** **2**).^[^ ^29a^ ^]^ Among a wide variety of natural materials that have been tested, a polysaccharide such as an alginate, glycosaminoglycans (GAGs) and chitosan are the most commonly used. Protein‐based materials, such as collagen and elastin, have been also studied in the development of CLTI targeted therapies as well as synthetic/recombinant materials.^[^ ^29b^ ^]^ Moreover, biomaterials offer a potent biotechnological solution to engineer synergic treatments to improve the available tools to induce tissue regeneration and angiogenesis when treating the complex pathophysiology of CLTI.^[^ ^29b,c^ ^]^

### 2.1 Biomaterials Functionalized with Growth Factors/Cytokines

As previously discussed, it is essential to improve the efficacy of growth factors by prolonging their half‐life and controlling the dose. Growth factor/cytokine‐loaded biomaterials represent a strategy to protect them from degradation. Additionally, by using biomaterial‐based vehicles allows to target the ischemic tissue specifically and to achieve a sustained and controlled release. This factor is critical in order to develop a mature and stable vascular network.^[^ ^13a^ ^]^

To date, a wide variety of naturally derived or synthetic materials have been used to deliver growth factors in preclinical models of CLTI. Alginate, chitosan, collagen, poly‐lactic‐co‐glycolic acid (PLGA), gelatin, fibrin, and dextran have been adopted in several types of formulations. Single‐component or complex biomaterial‐based approaches such as hydrogels, microspheres, and nanoparticles alone or in combination with a broad spectrum of pro‐angiogenic cytokines have been tested in the last decade (**Table** **1**).^[^ ^29^ ^]^

**Table 1:** CLTI preclinical trials with biomaterials/growth factors systems

Materials	Material forms	Growth factor/proteins	Animal	Model	Perfusion	Revascularization	Others	Refs.
Alginate	Hydrogel	HGF	Mouse (*n* = 10)	Excision of the femoral artery	Increased	Increased arterioles		^[^ ^33^ ^]^
Alginate		IGF+VEGF	Mouse (*n* = 10)	Ligation of iliac and femoral arteries and veins	Increased		Improved innervation, limb salvage Regeneration: increased CNFs, fiber diameters decreased apoptosis	^[^ ^34a^ ^]^
Alginate	Hydrogel	IGF+VEGF	Rabbit (*n* = 6–9)	Lateral circumflex artery, and femoral arteries ligation	Increased	Increased capillary density	Increased muscle fibers size	^[^ ^34b^ ^]^
Alginate	Hydrogel	IGF+VEGF	Mouse (*n* = 4–6)	Ligation of iliac and femoral arteries and veins	Increased	Increased capillary density		^[^ ^34b^ ^]^
Alginate‐PGLA	PGLA microspheres in alginate hydrogel	VEGF	Mouse (*n* = 7)	Femoral artery excision		Capillary and arteriole density PECAM‐I increase	Limb salvage	^[^ ^35^ ^]^
Alginate‐PLGA	PLGA microspheres +alginate hydrogel	VEGF+ ANGPT‐2	Mouse (*n* = 7)	Excision of the femoral artery		Increased capillaries and arterioles	Reduction of fibrosis	^[^ ^36^ ^]^
Alginate‐PLGA		HSP27 +VEGF	Mouse (*n* = 10)	Ligation of Iliac artery and excision of the femoral artery		Arteriole and capillary density	Limb salvage Reduced apoptosis	^[^ ^37^ ^]^
Alginate‐collagen	Alginate microspheres in collagen hydrogel	SDF‐1	Mouse (*n* = 9)	Single ligation of the femoral artery	Increased	Increased capillaries and arterioles density and diameters Pro‐angiogenic cytokines	Increased SDF‐1 receptor (CXCR4)	^[^ ^38^ ^]^
Chitosan‐HP	Hydrogel	FGF‐2	Rat (*n* = 8)	Femoral artery ligation	Increased	Capillary density increased		^[^ ^39^ ^]^
Collagen‐HP	Hydrogel	FGF‐2	Rabbit (*n* = 5)	Femoral artery excision		Capillary density increased	Oxygen perfusion increased	^[^ ^40^ ^]^
Dextran‐gelatin	Nanoparticles	VEGF	Mouse (*n* = 6)	Excision of femoral artery and branches	Increased	Capillary density increased		^[^ ^41^ ^]^
Dextran‐PLGA	Microspheres	VEGF	Rat (*n* = 12)	Excision of the femoral artery		Capillaries and Arterioles increased		^[^ ^42^ ^]^
Fibrin‐HP	Hydrogel	FGF‐2	Mouse	Ligation of the femoral artery		Increased vascularization	Reduction of Muscle fibrosis and inflammation	^[^ ^43^ ^]^
Fragmine/protamine	Nanoparticles	bFGF	Rabbit (*n* = 6)	Excision of the femoral artery		Increased collateral arteries		^[^ ^44^ ^]^
Fragmine/protamine	Nanoparticles	bFGF	Mouse (*n* = 10)	Excision of the femoral artery		Increased arterioles	Limb salvage Improved oxygen reperfusion	^[^ ^45^ ^]^
Gelatin	Hydrogel	FGFs	Rabbit (*n* = 8)	Excision of iliac and femoral arteries and collaterals	Increased	Increased vascular density		^[^ ^46^ ^]^
Gelatin	Hydrogel	FGFs + plasma‐derived GFs mixture	Mouse (*n* = 10)	Excision of iliac and femoral arteries and veins	Increased	Increased capillaries and arterioles		^[^ ^47^ ^]^
Gelatin	Microspheres	bFGF	Dog	Ligation of Iliac artery plus excision of the femoral artery	Increased	Increased capillaries and arterioles		^[^ ^48^ ^]^
Gold	Nanoparticles	VEGF	Mouse (*n* = 8)	Excision of iliac artery and vein and femoral artery	Increased	Increased capillaries		^[^ ^49^ ^]^
High‐density lipoprotein (HDL)	Nanodiscs	Substance P	Diabetic mouse (*n* = 12)	Femoral artery ligation	Increased	Increased capillaries and arterioles formation	Limb salvage Reduced fibrosis Immunomodulation: reduction of M2 macrophages	^[^ ^50^ ^]^
Liposomes		VEGF	Mouse (*n* = 4)	Iliac and femoral artery ligation			Increased blood vessel permeability	^[^ ^51^ ^]^
Peptide	Nanofibers	Substance‐P	Mouse (*n* = 6)	Excision of the femoral artery and side collaterals	Increased	Increased arterioles and capillaries mature vessels formation	MSCs recruitment Inhibition of fibrosis and cell apoptosis	^[^ ^52^ ^]^
Peptide	Nanofibers	VEGF mimetic	Mouse (*n* = 10)	Single ligation of the femoral artery	Increased	Blood vessels density increased		^[^ ^53^ ^]^
PLGA		VEGF	Mouse (*n* = 20)	Excision of iliac, femoral and collateral arteries and veins	Increased	Increased mature vasculature		^[^ ^54^ ^]^
PLGA	Nanoparticles	FGF‐2	Mouse (*n* = 4)	Occlusion of the saphenous artery		Increased number and diameter of arterioles		^[^ ^55^ ^]^

The most studied material systems in CLTI preclinical models so far have been hydrogels composed of alginate. Alginate is a widely use polymer due to its biocompatibility, and tunability to formulate hydrogels with suitable degradation and release profile, as well as adequate mechanical properties. When implanted into a murine CLTI model, HGF loaded‐alginate hydrogel resulted in increased blood perfusion and arteriole density over that of the HGF alone treated group.^[^ ^33^ ^]^ The injectable alginate biomaterial was designed to affinity‐bind heparin‐binding proteins establishing an interaction of alginate‐sulfate with HGF, resulting in its protection from degradation. Sustained release of HGF was additionally improved.^[^ ^33^ ^]^ The co‐delivery of insulin growth factor (IGF) and VEGF from the same hydrogel not only improved the vascularization but also had a beneficial effect on muscle fibers in both mouse^[^ ^34^ ^]^ and rabbit^[^ ^34b^ ^]^ models. The delivery VEGF and IGF showed a considerable improvement in effectiveness and safeness when compared with systemic injections of the free growth factors. In particular, an increase in fiber innervation, regeneration, diameter, limb salvage, and reduced apoptosis was observed in the mice while an increase of fiber diameter was observed in the rabbits.^[^ ^34b^ ^]^

Alternatively, PLGA microspheres have been incorporated into alginate hydrogel in a hybrid system to avoid over‐expression or overdose of the factors that could lead to undesired side effects. PLGA is an FDA approved polymer generally recognized as safe (GRAS) widely used for protein delivery. That has been used to deliver human recombinant VEGF alone^[^ ^35^ ^]^ or in combination with other proteins such as angiopoietin‐1 (ANG‐1)^[^ ^36^ ^]^ and heat‐shock protein 27 (HSP27).^[^ ^37^ ^]^ All of these approaches have improved vessel density in murine CLTI models. Additionally, PLGA/alginate/hrVEGF+ANG‐1 was observed to reduce muscle necrosis.^[^ ^36^ ^]^ The use of an hybrid system of VEGF‐loaded PLGA microparticles into an alginate hydrogel entrapping HSP27 leads to a sequential co‐delivery of both growth factor. Subsequently, the construct resulted in an increase of the number of arterioles and an improvement in limb salvation.

Key components of the extracellular matrix (ECM), such as collagen, elastin, and proteoglycans, have been widely used in tissue engineering, and CLTI is not an exception. ECM is the complex three‐dimensional environment that surrounds cells and orchestrates healthy development and homeostasis of tissues.^[^ ^56^ ^]^ ECM‐inspired materials attempt to mimic the ECM environment and therefore, have the purpose of providing chemical, mechanical, and biological inputs to cells of promoting regenerative programs in damaged tissues.^[^ ^3^ ^]^ An alternative formulation involved the fabrication of alginate microspheres and their incorporation in a collagen hydrogel, as a delivery system for the stromal cell‐derived factor‐1 (SDF‐1) chemokine that directs cell migration and the formation of blood vessels.^[^ ^38^ ^]^ This system based on microparticles into polymer matrix, prolonged the release of SDF‐1, while maintaining its bioactivity. When administered intramuscularly in a mouse CLTI model, this formulation induced an increase of capillaries and arterioles. Additionally, an upregulation of angiogenic cytokines and the activation of SDF‐1 receptors were reported.

Collagen and heparin (HP) based matrix was used without any crosslinking in order to achieve FGF‐2 sustained release over ten days. FGF‐2 was entrapped in the collagen matrix and tested in a rabbit CLTI model where it showed an increase in capillary density and improved oxygen reperfusion.^[^ ^40^ ^]^ FGF‐2 has also been loaded in other hybrid composites with conjugated HP. Indeed, collagen‐HP/FGF‐2^[^ ^39^ ^]^ and fibrin‐HP/FGF‐2^[^ ^43^ ^]^ hydrogels have both shown angiogenic potential in murine models of CLTI, as well as the ability to reduce fibrosis and inflammation in the case of HP/FGF‐2 hydrogel.

Finally, a hydrogel made of gelatin by polyionic complexing with essential fibroblast growth factor (bFGF) has been demonstrated as a promising delivery system for the protein. Indeed, while a gelatin‐bFGF hydrogel increased blood perfusion and arterioles in a rabbit,^[^ ^46^ ^]^ a gelatin hydrogel loaded not only with bFGF but also with a mix of plasma‐isolated growth factors^[^ ^47^ ^]^ obtained similar results in a mouse model. Gelatin has been tested not only in the form of a hydrogel but also in a microsphere format in a canine model. Gelatin microspheres crosslinked with glutaraldehyde were shown to prolong the release of bFGF.^[^ ^48^ ^]^ A gelatin hydrogel/microspheres/bFGF system is the only biomaterial formulation that has reached clinical application. Indeed, the formulation based on bFGF‐incorporated gelatin hydrogel microspheres through glutaraldehyde crosslinking demonstrated benefits in a phase I‐IIa improving CLTI patient symptoms^[^ ^30^ ^]^ when injected intramuscularly in ten patients. After 24 weeks, patients showed improvements in a distance walking test, in the rest pain scale and in the cyanosis development. These results suggest an increasing therapeutic relevance of using biomaterials systems to deliver growth factors that should encourage further clinical trials in CLTI patients.

A wide variety biomaterial different in nature (synthetic/recombinant/natural) has been used in the fabrication of microspheres, nanoparticles or nanofibers to deliver growth factors with promising performances in animal models. Dextran microparticles containing VEGF_165_ were encapsulated into PLGA microspheres and showed the ability to restore blood flow in a rat ischemia model.^[^ ^42^ ^]^ Similarly, gelatin nanoparticles from glycidyl methacrylate dextran synthesized through phase separation method, also produced a significant increase in blood flow perfusion and capillary density in a rabbit model.^[^ ^41^ ^]^ Besides, a microbubble contrast‐agent‐based system was used to improve the delivery of PLGA nanoparticles loaded with FGF‐2, showing angiogenic potential in a murine CLTI model.^[^ ^55^ ^]^

Another strategy utilized Fragmin/protamine microparticles for the release of platelet‐rich plasma (PRP)^[^ ^44^ ^]^ or FGF‐2.^[^ ^45^ ^]^ Protamine neutralizes Fragmin forming a complex that lacks anticoagulant activity. Indeed, Fujita et al. prepared the microparticulate system by adding protamine dropwise into a Fragmin solution, to deliver PRP, resulting in an increased collateral sprouting and calf blood pressure in a rabbit model of CLTI.^[^ ^44^ ^]^ Fragmin/protamine microparticles loaded with FGF‐2 were prepared by the same technique, and showed similar results in a mouse model in limb rescue and increased oxygenation.^[^ ^45^ ^]^

Liposomes and HDL nanodiscs have also been tested in combination with growth factors, thanks to their ability to target the cell membrane. In order to avoid surgical implantation or multiple local injections, liposomes with medium size of <200 nm were engineered to display a VEGF mimetic epitope. A sequential approach involving two intravenously administered systems was proposed. First, VEGF‐loaded liposomes increased blood vessel permeability for seven days, allowing the second liposomes to accumulate into the ischemic sites via enhanced permeation and retention (EPR) effect in a mouse model.^[^ ^51^ ^]^ HDL nanodiscs loaded with substance P were develop to increase its half‐life and therapeutic efficacy. Substance P half‐life was improved >1000‐fold consequently improving its retention in the bone marrow. This accumulation finally leads to a sustained release of substance P that benefits not only in vascularization but also in reduced fibrosis, necrosis, and apoptosis.^[^ ^50^ ^]^ Delivery of growth factors loaded into nanoparticulated systems use the “tumor‐targeting strategy.” Furthermore, VEGF‐conjugated gold nanoparticles with a size around 27 nm were prepared by citrate‐reduction of Au^3+^, demonstrating a sustained release and evoked stable angiogenesis and limb reperfusion.^[^ ^49^ ^]^

Finally, peptide nanofibers have also demonstrated their potential as protein release systems. Self‐assembling peptides showed attractive properties for regenerative medicine forming nanometric fibers (<10 nm) that self‐organize into 3D ECM‐like structures. Self‐assembled peptide hydrogels were developed by mixing RADA16‐II (AcN‐RARADADARARADADA‐CONH2) and RADA‐SP (AcNRARADADARARADADAGGRPKPQQFFGLM‐CONH2) developing stable b‐sheets into a fibrous nanostructure. The peptide‐based systems showed to recruit cells, promote neovascularization, and inhibit apoptosis and fibrosis in a murine model.^[^ ^52^ ^]^ Furthermore, nanofibers engineered with VEGF mimetic peptides improved perfusion and in increased blood vessel density in a murine model. A VEGF mimetic peptide called SLanc, which was anchored to a self‐assembling multidomain peptide, has been developed. SLanc peptide amphiphiles formed quickly injected hydrogels, while being able to activate and upregulate angiogenic receptor expression.^[^ ^53a^ ^]^ Furthermore, VEGF‐mimicking peptide KLTWQELYQLKYKGI‐NH2 was designed to promote self‐assembly into cylindrical nanoarchitectures.^[^ ^53a^ ^]^ After treatment with both systems, functional recovery was observed related to the VEGF‐mimetic behavior.

Preclinical trials have shown improved angiogenesis, tissue regeneration of the biomaterial‐growth factor system over that of the pure growth factors administration. The performance of these systems suggests that the use of a biomaterial delivery system is essential in prolonging the efficacy of growth factors that results in the restoration of stable blood circulation with therapeutic relevance. These studies were conducted in the range of 14–28 days after administration. However, most of these engineered biomaterials act mainly as vehicles in order to improve the delivery or to protect the growth factor in the mid‐long term, without adding any additional bioactivity to the therapy.

Nevertheless, biomaterials offer the tools to engineer complex systems for co‐delivery of various growth factors or to combine them with other strategies. An exciting example will be the engineering of new mimetic peptides able to enhance growth factor performance provides an exciting strategy that offers high tunability to overcome growth factor drawbacks. Efficacy of these biomaterial‐growth factor systems, as well as the protein release profile, needs to be confirmed and validated at the clinical level.

### 2.2 Biomaterials Functionalized with Nucleic Acids

Gene therapy consists of the administration of nucleic acids, such as DNA, small interfering RNA (siRNA), or microRNA (miRNA) to modify a specific gene function when targeting a disease.^[^ ^57^ ^]^ Gene therapy employs the cell encoding apparatus to stably produce or suppress active biomolecules, allowing the targeting of specific genetic dysfunction, and the avoidance of frequent drug administration. Gene therapy is one of the most explored novel therapeutic strategies to promote angiogenesis in CLTI patients. The focus has been predominantly on plasmid DNA (pDNA) encoding pro‐angiogenic growth factors such as VEGF_121_, _165_, FGF‐2, and HGFs that have reached the clinical experimental stage. The results of these clinical trials demonstrated the safety and the potential of these strategies. Indeed, an improvement in patient symptoms such as ulcers healing, tissue oxygenation, and limb reperfusion has been reported.^[^ ^58^ ^]^ However, gene therapy presents limitations such as restricted transgene expression due to deficient in vivo transfection efficacy and high degradation rate. These drawbacks make it necessary to design and develop more efficient nucleic acid transfer systems.

To address this need, biomaterial‐based nucleic acid carriers have been developed and tested in preclinical CLTI models. The purpose of these biomaterial carriers is to increase gene therapy efficacy by protecting the DNA/RNA from degradation by the nucleases, uptake from phagocytes, as well as to target specific tissue/cell sites, and to facilitate cell internalization.^[^ ^59^ ^]^ To this end, several polymeric materials mostly in the form of nanoparticles/liposomes, but also as hydrogel/microspheres system, have been tested in preclinical CLTI models and have shown encouraging and promising results (**Table** **2**).

**Table 2:** CLI preclinical trials with biomaterials/nucleic acid systems

Materials	Material forms	Genes/nucleic acid	Animal	Model	Perfusion	Revascularization	Others	Refs.
Elastin‐like polypeptide	Hydrogel + Microspheres	pDNA eNOS+ IL‐10	Mouse	Single ligation of femoral artery and vein	Increased	Increased capillary density and pro‐angiogenic cytokines genes	Decreased Inflammatory cells	^[^ ^60^ ^]^
PDAPEI synthetic polymer	Particles	pDNA VEGF	Mouse (*n* = 5)	Ligation of the iliac and the femoral arteries	Increased	Vessel density increased		^[^ ^61^ ^]^
Magnetic DNA‐gelatin	Nanospheres	pDNA VEGF	Rabbit (*n* = 6)	Excision of femoral artery and branches	Increased	Capillary density increased		^[^ ^62^ ^]^
PEG	Liposomes	pDNA bFGF	Mouse (*n* = 4–6)	Single ligation of the femoral artery	Increased	Increased capillary density		^[^ ^63^ ^]^
PLGA	Nanoparticles	pDNA VEGF	Mouse (*n* = 5)	Total excision of femoral artery		Increased capillary density	Increased expression of VEGFs in vivo	^[^ ^64^ ^]^
PEG and DOTAP	Liposomes	pDNA (bFGF)	Mouse (*n* = 5−6)	Single ligation of femoral artery	Increased			^[^ ^65^ ^]^
Bubble	liposomes	miR‐126	Mouse (*n* = 4–6)	Single ligation of femoral artery	Increased	Secretion of pro‐angiogenic factors		^[^ ^66^ ^]^
Heparin‐PEI		pDNAVEGF165	Mouse (*n* = 8–10)	Ligation of iliac and femoral artery	Increased	Capillary density increased		^[^ ^67^ ^]^
PLGA	Nanoparticles	miR‐126	Mouse		Increased	Capillaries and arterioles increased		^[^ ^68^ ^]^

PLGA nanoparticles, prepared by the traditional method of double emulsion–solvent evaporation, have been used to deliver VEGF plasmid, showing an average size of 201 nm and >87% entrapment efficiency. Therefore, pDNA sustained release for 11 days in a mouse model of CLTI was reported, resulted in increased expression of VEGFs. Consequently, a marked capillary formation increases.^[^ ^64^ ^]^ Similarly, the synthetic polycationic polymer Polydiacetylene‐polyethylenimine (PDAPEI) has been used to engineer particles with an average size of 160–250 nm, carrying VEGF‐A as pDNA. The PDAPEI/pDNA polyplex nanoparticles were able to induce blood perfusion in an ischemic mouse model.^[^ ^61^ ^]^ pVEGF_165_ has also been delivered with hybrid gelatin‐magnetic DNA nanospheres with an average size ranging from 5 to 20 nm. Indeed, these magnetic spheres markedly increased the blood perfusion and vessel density after being magnetically directed at the ischemic site in a rabbit CLTI model.^[^ ^62^ ^]^ Furthermore, the formulation heparin‐PEI‐VEGF plasmid was designed in order to improve transfection efficiency, strongly inhibited when using cationic polymers. The incorporation of heparin leads to a significantly higher efficiency that consequently stimulates the growth of arterioles and capillaries in a mouse ischemia model.^[^ ^67^ ^]^

Other growth factors‐encoding pDNA has been examined in combination with liposomes and tested in CLTI models. Liposomes are often used to deliver a DNA cargo, thanks to their ability to target and fuse with the cell membranes. Polyethylene glycol (PEG) modified bubble liposomes, entrapping ultrasound gas and composed of 1,2‐dipalmitoyl‐sn‐glycero‐3‐phosphocholine (DPPC) and 1,2‐distearoyl‐sn‐glycero‐3‐phosphatidyl‐ethanolamine‐polyethyleneglycol (DSPE‐PEG2000‐OMe), were prepared by a reverse‐phase evaporation method.^[^ ^63^ ^]^ Additionally, same authors engineered PEG‐1,2‐dioleoyl‐3‐trimethylammonium‐propane (DOTAP) liposomes.^[^ ^65^ ^]^ Both liposome formulations were used to deliver pFGF in a mouse model of CLTI having the ability to induce an increase of blood flow reperfusion. Besides, the injection of PEG‐modified liposome increased capillary density.

Most of the formulations tested involved the delivery of a single gene that targets angiogenesis. Consequently, besides the improvement in the delivery and protection of the nucleic acid, present similar limitations than the viral vectors in terms of therapeutic performance. A previous study in our group has used an elastin‐based hydrogel/microsphere system for dual gene delivery of endothelial nitric oxide synthase (eNOS) and interleukin‐10 (L‐10), targeting both angiogenesis and inflammation.^[^ ^60^ ^]^ This system was able to induce angiogenesis and attenuate inflammation in CLTI model, proving the importance of targeting multiple molecular pathways in the disease. Indeed, further studies involving the co‐delivery of genes that target not only angiogenesis and inflammation but also oxidative stress and muscle fiber regeneration are required.

As well as gene delivery, the administration of miRNA has been investigated for therapeutic angiogenesis. Indeed, miRNAs have recently been identified as regulators of gene expression involved in several angiogenic processes, such as the proliferation and the migration of endothelial cells.^[^ ^69^ ^]^ Among the angiogenesis‐related, miRNA‐126^[^ ^70^ ^]^ and miRNA‐132^[^ ^71^ ^]^ have been well‐described to modulate angiogenesis by inhibiting negative regulators and consequently enhancing growth factor signaling. PLGA nanoparticles containing perfluoro‐1, 5‐crown ether (PFCE), detectable with magnetic resonance imaging (MRI), were used to transfect endothelial cells with miRNA‐132. When injected in a mouse model of CLTI, were able to improve limb reperfusion due to threefold increase of the endothelial cells survival in vivo, in addition to a 3.5‐fold improvement of blood perfusion to ischemic limbs.^[^ ^72^ ^]^ miRNA‐126 has been complexed both with liposomes^[^ ^66^ ^]^ and PLGA particles.^[^ ^68^ ^]^ The administration of both formulations in a CLTI model resulted in improvement of blood reperfusion and an increase in capillary numbers. Taken together, this evidence highlights the promising role of the biomaterial/miRNA combination in inducing therapeutic angiogenesis and offers a sophisticated method of regulation. In summary, preclinical trials have repeatedly shown the potential of biomaterials as an efficient adjuvant of gene therapy for CLTI.

Further investigation on the possible mechanisms and the long‐term effect of these therapies in vivo, assessing toxicity, inflammation and fibrosis, is required before proceeding to the clinical phase.

### 2.3 Biomaterials for Extended Cell Therapy

Cell therapy is an attractive strategy to stimulate therapeutic angiogenesis for several reasons.^[^ ^73^ ^]^ First, transplanted cells can be a stable reservoir of multiple pro‐angiogenic growth factors that can induce the formation of stable vessels. The release of these growth factors and other cytokines can also be balanced and physiologically relevant since this release is a consequence of the cell‐response to the pathological environment. Furthermore, stem cells can differentiate into vascular cells, providing direct structural support for angiogenesis by integrating into the existing vasculature wall.^[^ ^73a^ ^]^ Moreover, the use of autologous cells prevents the problem of host immune rejection.^[^ ^73b^ ^]^

Somatic stem and progenitor cells including bone marrow mononuclear cells (BMMNCs),^[^ ^74^ ^]^ endothelial progenitor cells (EPCs),^[^ ^75^ ^]^ and mesenchymal stem cells (MSCs)^[^ ^76^ ^]^ have been investigated in several CLTI clinical trials, revealing potential efficacy and safety.^[^ ^8^ ^]^ In particular, the intramuscular administration of BMMNCs markedly increased the ankle‐brachial index and the oxygen pressure, and improved ulcer healing.^[^ ^77^ ^]^ Besides, the intravascular injection of MSCs was able to improve the rates of revascularization and oxygenation.^[^ ^78^ ^]^

Despite the encouraging therapeutic effects shown in preclinical and clinical trials, cell therapy still presents several challenges, including limited cell retention and survival after the injection.^[^ ^8^ ^]^ Indeed, the hypoxic and inflammatory environment of CLTI does not favor cell survival since it induces apoptosis or clearance by immune cells in the early days after administration. Multiple injections do not improve the short half‐life of mononuclear cells in the ischemic muscle, and can also cause excessive inflammation.

Biomaterials offer new tools that can improve the efficacy of stem cells by different strategies. First, the encapsulation of cells into hydrogels to provide ECM clues that mimic the cellular niches, and protect them while stimulating their paracrine capacity.^[^ ^79^ ^]^ On the other hand, the use of nanometric vehicles as transfection vectors to genetically manipulate the cells to produce a particular growth factor to be more resistant to apoptosis and oxidative stress.^[^ ^29c^ ^]^

#### 2.3.1 Biomaterials Matrices for Cell Encapsulation

The use of ECM‐derived matrices for cell encapsulation has been widely explored in recent years. Indeed, these matrices not only protect cells from the host pathological environment but also mimic the natural niche that primes proliferation, differentiation, and secretion of tissue remodeling factors.^[^ ^79^, ^80^ ^]^ Therefore, the use of ECM‐based matrices provides mechanical end structural support and activates cell‐signaling through cell–matrix interactions to guide the vascular assembly.^[^ ^81^ ^]^ Furthermore, these matrices can be used as scaffolds that maintain and stabilize the cell culture before transplantation.

Several biomaterial/cell systems have been tested in preclinical CLTI models showing promising revascularization effects (**Table** **3**). A wide variety of natural and synthetic materials such as hyaluronic acid (HA), collagen, gelatin, alginate, specific‐peptides, or polyesters have been successfully used.^[^ ^82^ ^]^ Also, different cells with key roles in angiogenesis such as endothelial cells, stem cells, and macrophages were used as the bioactive cargo.^[^ ^83^ ^]^ The first biomaterial/cells system tested in a CLTI model was composed of an HA solution, and human umbilical vein endothelial cells (HUVECs).^[^ ^84^ ^]^ This combination showed the ability to improve reperfusion and angiogenesis in the limb by prolonging cell retention, survival, and by promoting the engrafting of HUVECs into the endothelium. Recently, Ludwisky et al.^[^ ^82a^ ^]^ encapsulated a pro‐angiogenic type of macrophage (expressing Tyrosine‐protein kinase (Tie‐2) receptor for the pro‐angiogenic factor Angiopoietin‐2) in an alginate hydrogel and demonstrated the beneficial effect on tissue reperfusion and vascularization in a mouse CLTI model. Alginate capsules of 300 µm were prepared by a reproducible GMP‐compliant method for macrophages delivery. Furthermore, the encapsulation of the macrophages substantially increased cell retention when compared with injected naked cells.^[^ ^82a^ ^]^

**Table 3:** CLI preclinical trials with biomaterials/cells systems

Materials	Material forms	Cells	Species/sample size	Model	Perfusion	Revascularization	Others	Refs.
Alginate	Hydrogel	Tie‐2‐expressing macrophages	Mouse (*n* = 15)	Single ligation of the femoral artery	Increased	Increased arterioles Increased capillaries	No effect on inflammation, apoptosis and muscle damage	^[^ ^82a^ ^]^
C7 engineered protein + 8‐arm PEG‐P1 protein + PNIPAM and RGD sequence	Hydrogel	iPSCs‐ECs	Mouse (*n* = 9–14)	Excision of the left femoral artery		Capillaries and arterioles increased	Enhanced cell retention	^[^ ^85^ ^]^
Collagen	Porous scaffold	BM‐MSCs	Rabbit (*n* = 15)	Femoral artery excision	Increased oxygen saturation ratio	Increased arterioles and capillaries		^[^ ^86^ ^]^
CS‐IGF‐1C C‐domain peptide of insulin‐like growth factor‐1	Hydrogel	hP‐MSCs	Mouse (*n* = 5)	Ligation of Iliac artery		Capillaries and arterioles increased	Activation of VEGFR pathway	^[^ ^87^ ^]^
GFFYK peptide	Hydrogel	MSCs	Mouse (*n* = 6)	Unilateral femoral artery ligation and excision	Increased	Increased arterioles and capillaries	Reduced inflammatory cell infiltration Reduced collagen deposition Limb salvation	^[^ ^88^ ^]^
HA		HUVEC	Mouse (*n* = 5)	Femoral and iliac artery ligation and excision	Increased	Increased arterioles and capillaries	Increased cell survival, and secretion activity Engraft of the cells in the blood vessels	^[^ ^84^ ^]^
PEGylated fibrin	Hydrogel	MSCs	Rat	Excision of femoral artery		Increased number of mature blood vessels		^[^ ^89^ ^]^
Platelet lysate	Hydrogel	MSCs	Mouse (*n* = 4)	Femoral artery excision	Increased			^[^ ^90^ ^]^
Poly(ethylene glycol)‐poly(amino ketal) (PEG‐PAK)	Micelles	SDF‐1*α* transfected hADSCs	Mouse (*n* = 8)	Ligation iliac arteries and braches and excision of femoral artery	Increased	Increased capillaries and arterioles	Increase of I‐CAM and V‐CAM expression	^[^ ^91^ ^]^
Poly(*β*‐amino esters) (PBAE)	Nanoparticles	VEGF‐transfected HUVEC	Mouse (*n* = 10)	Ligation iliac arteries and braches and excision of femoral artery		Engraftment of HUVEC into the vasculature Increased vessel density	Limb salvage	^[^ ^92^ ^]^
Polycaprolactone (PCL)/gelatin scaffolds	Electrospun scaffold	iPSCs‐ECs	Mouse (*n* = 5)	Subcutaneous	Increased	Increased capillaries and arterioles	VEGF expression increase Reduction of the inflammatory response	^[^ ^93^ ^]^

The most studied cells in the context of biomaterial encapsulation for CLTI application are the MSCs. MSCs are multipotent stem cells present in the bone marrow, umbilical cord blood, and the placenta. These cells can differentiate into several types of cells—osteoblasts, chondrocytes, cardiomyocytes, and endothelial cells—when supported with appropriate chemical/biological/physical cues.^[^ ^77^, ^94^ ^]^ Consequently, MSCs have been encapsulated in different biomaterials for CLTI management.^[^ ^85^, ^87^, ^88^, ^90^, ^91^, ^92^, ^93^, ^95^, ^96^ ^]^ A construct of autologous bone marrow stem cells (BMSCs), seeded in a collagen solution forming a porous scaffold, was able to induce the formation of mature vessels and oxygen saturation in a preclinical model. Collagen acted not only as a cellular delivery system but also as an angiogenic inducer by itself, showing a better effect than BMSCs alone.^[^ ^86^ ^]^ Our laboratory has fabricated a tunable type‐I collagen microgel platform that was able to modulate the paracrine angiogenic action of human mesenchymal stem cells (hMSCs).^[^ ^97^ ^]^ The microgels were prepared by crosslinking with 4‐armPEG and optimized by varying polymer concentration, collagen‐crosslinking ratio as well as embedding cell densities. Cells embedded into the microgel were able to induced angiogenesis, ambulation improvement, reduce inflammation and necrosis in a mice model of CLTI.^[^ ^97^, ^98^ ^]^ A more in‐depth molecular analysis to understand the influence of macromolecular concentration on the paracrine phenotype of hMSCs revealed that the interaction of collagen I with hMSCs’ integrins was crucial in the secretion of pro‐angiogenic mediators. Moreover, collagen microgels significantly improved the performance of similar cell‐delivery platforms with a 20‐fold lower cell dose, as well as allowed to elucidate a mechanism involving pathways modulated in vivo.^[^ ^97^ ^]^

Fibrin is another ECM‐protein that has been used for the development of BMSCs delivery systems. A fibrin‐based hydrogel was engineered by combining difunctional succinimidyl glutarate PEG with human fibrinogen and thrombin to promote the crosslinking. PEGylated fibrin showed a significant improvement as a support for MSCs in a rat model while increasing the number of mature blood vessels after treatment.^[^ ^89^ ^]^

In addition, synthetic peptide‐based small molecular hydrogels have been used in combination with MSCs. A novel small molecule hydrogel, named as Nap‐GFFYK‐Thiol, has been recently synthesized via a disulfide bond reduction method. The synthetic peptide hydrogel performed as a favorable niche to promote cell survival, as well as promoting the secretion of pro‐angiogenic factor.^[^ ^88^ ^]^ The new system was able to induce revascularization, angiogenesis, and cause a decrease in inflammatory cell infiltration and collagen deposition.^[^ ^88^ ^]^ Furthermore, a hybrid construct made of C‐domain of insulin‐like growth factor‐1 (IGF‐1C)‐chitosan was used to deliver MSCs in a mouse model of CLTI, showing improved revascularization due to the activation of the receptors for VEGFRs pathway.^[^ ^87^ ^]^ Another example of an ECM‐based system is a novel fibrinogen‐rich platelet lysate recently developed to deliver MSCs. The platelet rich hydrogel showed mechanical properties similar to pure fibrin hydrogels while providing a nutritive environment to the MSCs that promote their differentiation and survival in the long term.^[^ ^90^ ^]^

Synthetic materials such as hydroxiapatites have been used to successfully encapsulate stem cells. Such is the case of hydroxyapatite coated poly(l‐lactic acid) (PLLA) microspheres, that were generated an injectable cell scaffold. The nanoscaffolds, that were used as a carrier for BMMNCs, were injected into the ischemic muscle.^[^ ^95^ ^]^ The system induced an increase of capillaries and arterioles that were correlated with the upregulation of the pro‐angiogenic cytokines VEGF and bFGF.^[^ ^95^ ^]^

Recently, induced pluripotent stem cells (iPSCs) have been encapsulated into a biomaterials carrier and have shown angiogenic potential in both a subcutaneous and a CLTI models.^[^ ^85^, ^93^ ^]^ The main goal of the new formulations is to overcome the main limitation of transplanted cell death. Among stem cells, iPSCs offer the possibility to reprogram pluripotency from adult human cells,^[^ ^96^ ^]^ overcoming the problem of immune‐rejection and opening for the future a patient‐specific therapy. In particular, Tan et al.,^[^ ^93^ ^]^ delivered induced pluripotent stem cell‐derived endothelial cells, (iPSCs‐ECs) using polycaprolactone (PCL)/gelatin electrospun scaffold. The parameters of the scaffold were engineered in order to improve cell survival that led to better blood perfusion when injected subcutaneously in a mouse model. Furthermore, Forster et al., designed a 3D injectable peptide hydrogel with dynamic control of mechanical properties.^[^ ^85^ ^]^ Consequently, the matrix was able to mechanically protect iPSC‐ECs during injection and promote long‐term iPSC‐EC retention. This rational design showed that a thermoresponsive recombinant protein hydrogel containing the RGD amino acid sequence in combination with iPSCs‐ECs was able to induce an increase in arterioles and capillaries density.

The development of biomaterial‐based systems to improve cell‐based therapies efficacy and reproducibility is still a need in tissue engineering.^[^ ^99^ ^]^ An engineered microcryogel systems has been developed to overcome these limitations.^[^ ^100^ ^]^ These microcryogels formed 3D microniches that efficiently protected cells from the mechanical forces through injection.^[^ ^101^ ^]^ Microcryogels exhibited enhanced mechanical properties when compared with microscale hydrogels which not only protect the cells during the process but additionally overcome most of the significant challenges of in vivo cell therapy.^[^ ^102^ ^]^ One example is the use of gelatin microcryogels which were engineered as 3D microniches as a strategy to enhance the therapeutic efficacy with a lower cell dosage for treating CLI in the mouse model.^[^ ^103^ ^]^ These gels were fabricated by cryogelation with a predefined shape and size. These microsystems exhibited high porosity which facilitated cell loading showing improved therapeutic performance with ten times less dosage than previously reported for CLI cell therapy.

Overall, these studies demonstrate the therapeutic potential of the biomaterials/cells constructs for CLTI and the ability of biomaterials to support and enhance cell therapy.^[^ ^99^ ^]^ Our study though is the only one that investigates the mechanisms of actions.^[^ ^97^ ^]^ More mechanistic studies are necessary to obtain a molecular profile that would help in the optimization of the therapeutic formulations. Comparative studies are necessary to select the optimal biomaterials/cell system to obtain therapeutic angiogenesis.

#### 2.3.2 Biomaterial as Transfection Vectors for Engineering Cells

One of the main drawbacks when using stem cells alone in order to promote angiogenesis, is the insufficient expression of angiogenic factors, as well as low cell viability after transplantation. While hydrogels/ matrices are mostly used to protect and enhance cell paracrine phenotype and differentiation capacity, nanoparticles or micelles are adopted to transfect and engineer the cells to obtain the desired functionality.^[^ ^28^ ^]^ Specifically, nanomaterials are used as non‐viral vectors for cell transfection to reprogram cells. Subsequently, a higher expression of angiogenic factors, increased survival, and higher resistance to apoptosis and inflammatory stimuli are potentially induced characteristics. In this field, the types of nanomaterials, the gene functionality transferred, and the recipient type of cells are varied.

Yang et al. used biodegradable poly (*β*‐aminoester) (PBAE) nanoparticles to transfect VEGF_165_ plasmid into MSCs.^[^ ^104^ ^]^ These VEGF_165_ over‐expressing cells injected intramuscularly in a murine CLTI model induced a notable increase in vascular densities and, a decrease in muscle necrosis and tissue fibrosis compared to that of the control.^[^ ^104^ ^]^ Similarly, biodegradable PBAE nanoparticles were used as vehicles to modify HUVEC genetically. Genetically transfected cells significantly enhanced VEGF expression preventing mice with hind limb ischemia from undergoing necrosis and limb loss.^[^ ^92^ ^]^

Additionally, micelles were used to transfect stem cells. For example, poly (ethylene glycol)‐poly (amino ketal) (PEG‐PAK) micelles were used to transfect human adipose‐derived stem cells (hADSCs) with stromal cell‐derived factor‐1 alpha (SDF‐1*α*) gene. SDF‐1 is a natural cytokine that mediates the homing of cells in inflammatory conditions.^[^ ^105^ ^]^ The SDF‐1 transfected cells presented a significant increase in SDF‐1*α* and VEGF expression that subsequently reduced apoptotic activity. Furthermore, the achieved growth factor overexpression induced an increase in revascularization and blood perfusion in a murine CLTI model.^[^ ^91^ ^]^ Cells can also be engineered to be resistant to oxidative stress (ROS). The presence of ROS in the ischemic tissue has been described as one of the main factors that minimize survival of engrafted cells. Chitosan is a family of natural polymers with an analogous structure to GAGs, which exhibits interesting physicochemical and biological properties for tissue engineering. The use of a chitosan hydrogel crosslinked with *β*‐glyceryl phosphate conjugated to cells before being implanted showed the ability of scavenge ROS in the ischemic tissue and thus increase transplanted cell survival in a CLTI model.^[^ ^106^ ^]^

Cells can be genetically engineered/manipulated not only through the delivery of genes but also by using miRNA. These noncoding RNAs, negatively regulate the translation of messenger RNA (mRNA) into protein for the purpose of inhibiting target genes at the therapeutic level.^[^ ^107^ ^]^ Nanoparticles also have application as miRNA carriers.^[^ ^72^ ^]^ Gomes et al. designed multifunctional nanoparticles that can not only deliver the pro‐survival/angiogenic miRNA (miR132 and miR424) but can also be used for cell MRI‐tracking in the tissue. These nanoparticles showed 50–90% transfection efficiency into endothelial cells. Endothelial cells engineered with NP‐miRNAs increased not only the cell survival but also, remarkably, the blood perfusion of ischemic mouse limb, if compared with the not‐transfected cell treatment group.^[^ ^72^ ^]^

Furthermore, a nanoscale material has also been used to direct the cells to the specific target zone in the tissue. Magnetic polystyrene‐copolymer nanoparticles containing iron oxide demonstrated the ability to guide EPCs to ischemic tissue when a magnetic field was applied. Furthermore, increased blood reperfusion was also observed.^[^ ^108^ ^]^ Moreover, EPCs transfected with magnetic nanoparticles isolated from the bacteria *Magnetospirillum magneticum* were recruited at the ischemic site, and this resulted in improved reperfusion of blood flow.^[^ ^108^ ^]^

In summary, reported results to date suggest that the use of biomaterials for cell engineering not only enhances cell functions such as cell viability or paracrine activity. Indeed, it can be used for precise cell targeting, homing and tracking. To improve non‐viral gene delivery further research on the cell‐material interface influence is necessary. The physicochemical properties of the material can have a separate or adjuvant role to modulate cell behavior that will subsequently improve cell transfection. Most of the materials used to engineer new biomaterial‐based transfection vectors mimic ECM cues that modulate cellular behaviors influencing transfection efficiency. Mechanistic studies to clarify this role are also needed to move forward in order to design next‐generation materials. Nevertheless, further comprehensive preclinical investigations as to the safety and cytotoxicity of these methods are required. Indeed, most of the studies mentioned above do not examine the possible in vivo side effects of these biomaterials carriers nor the long‐term effects on cellular viability and function.

### 2.4 Biomaterials for Exosome Delivery

According to recent findings, exosomes mediate the paracrine action of stem cells. Exosomes are nano‐sized structures (40–150 nm) that, due to their cell‐originated nature, are attracting attention as pharmacological tools.^[^ ^109^ ^]^ Exosomes play vital roles in intercellular communication and present the desired characteristic to act as delivery systems.^[^ ^25^ ^]^

Despite its potential as a therapeutic, exosome injection has shown a rapid clearance from circulation, which is a cause of concern. Additionally, exosomes are challenging to produce in large quantity while therapeutic doses needed are usually high. This indicates the need for a sustained delivery system to protect the encapsulated exosomes and prevent them from being cleared prematurely.^[^ ^110^ ^]^

There are a few examples of biomaterials that are used as exosome delivery systems targeting ischemic tissues for CLTI. However, the possibility of inducing tissue regeneration utilizing cell‐derived exosomes is promising. A chitosan thermosensitive hydrogel prepared *β*‐glycerophosphate as a crosslinker, was used to incorporate human placenta‐derived MSC‐derived exosomes.^[^ ^111^ ^]^ The injectable carrier enhances exosomes stability and attenuates their effect as well as their retention in vivo.

The tunability of biomaterial based formulations in terms of degradability and shape would potentially allow customizing exosome delivery systems. Even though is a young field of research the development of injectable delivery system that capable to efficiently deliver exosomes in the long term is highly appealing.^[^ ^112^ ^]^

### 2.5 Nitric Oxide Delivery Materials

Nitric oxide (NO) is an endogenous factor in cardiovascular homeostasis that plays a key modulatory role in various molecular targets necessary for ischemic vascular remodeling.^[^ ^113^ ^]^ Additionally, NO has shown the ability to favor the expression of pro‐angiogenic cytokines and growth factors in MSCs.^[^ ^114^ ^]^ Despite its therapeutic potential, its utilization is limited due to short half‐life and the lack of effective delivery strategies that limit its angiogenic effect in vivo. Consequently, systems that allow a sustained and controllable deliverable are needed for its application.^[^ ^115^ ^]^ NO has been used as a therapy resulting in modifications in cell function and phenotype, as well as inducing growth factor secretion through exosomes.

A chitosan NO‐releasing polymer designed as combinate‐shaped NO‐delivering compound of NO donors conjugated to the polymer was recently used to stimulate exosome release from MSCs.^[^ ^116^ ^]^ Previous studies from the same group showed that an NO‐releasing hydrogel delivering MSCs was able to promote angiogenesis while ameliorating heart function.^[^ ^117^ ^]^ The efficacy of the NO‐induced secreted exosomes was evaluated in a hind limb ischemia model with transgenic mice. Based on a similar approach, a hydrogel of chitosan‐NO donor was developed through conjugating *β*‐galactose caged NO donor to the polymer backbone. Furthermore, the engineered hydrogel presented the ability of NO release in the presence of *β*‐galactosidase.^[^ ^118^ ^]^ The formulation showed the potential of could stimulating cell differentiation from without the addition of exogenous growth factors by NO sustained delivery into the cellular microenvironment.

A novel NO system for precise delivery into disease sites was designed by using an engineered *β*‐galactosidase. The designed delivery system, named “MeGal‐NO”, improved its circulation stability and releases NO specifically mediated by the catalytic action of, a *β*‐galactosidase mutant from *Thermus thermophiles*..^[^ ^119^ ^]^ The use of a modified monosaccharide was used as a strategy to protect the designed Gal‐NO from undesired hydrolysis. Furthermore, NO targeted delivery significantly reduced undesired side effects caused by its systemic release and enhanced its therapeutic efficacy.

A recent study, showed an engineered gelatin hydrogel crosslinked by microbial transglutaminase. The enzymatic crosslinking led to ammonia deposition and, due to its partial oxidation, to the consequent release of NO.^[^ ^120^ ^]^ The NO delivery system displayed an elastic modulus of 1.5‐kPa offering a suitable environment to the hMSCs. This new platform was the first of its kind to elucidate hMSC source‐dependent angiogenic mechanisms. Endogenous NO plays a crucial role in the vascular formation and remodeling as well as an inflammatory modulator. In that sense, to design new NO donors with improved stability, long‐term NO release as well as improved biocompatibility can provide the necessary solutions to overcome current concerns of NO‐delivery systems efficacy. The rationale design of NO biomaterials represents a blossoming field of research to overcome drawbacks associated with therapeutic strategies targeting ischemic diseases.

## 3 Reinventing Biomaterials

In the last decade, the administration of biomaterial based matrices as a therapeutic has become attractive because of their ease in translation and their ability to influence cellular behavior in health and disease. In particular, ECM‐derived/based materials have shown regenerating and healing potential in animal models of CLTI due to the key role of this polymer in development and homeostasis.^[^ ^29^, ^121^ ^]^ Indeed, biomaterials alone have shown the capability of repairing the ischemic tissue by promoting angiogenesis, revascularization and muscle regeneration. Specifically, natural materials such as fibrin and decellularized ECM hydrogel have been investigated in CLTI preclinical models (**Table** **4**).

**Table 4:** CLTI preclinical trials with biomaterials systems alone

Materials	Material forms	Species/sample size	Model	Perfusion	Revascularization	Others	Refs.
Heparan Sulfate	Solution	Mouse (*n* = 8)	External iliac artery	Increased			^[^ ^122^ ^]^
Chitosan‐fibrin	Hydrogel	Mouse (*n* = 4)	Excision of iliac femoral artery	Increased	Increased capillary density	Limb salvage	^[^ ^123^ ^]^
Decellularized muscle ECM	Hydrogel	Rat (*n* = 3–4)	Excision of femoral artery	Increased	Increased arteriole and capillary density	Increased myoblast determination protein 1 (MyoD) + muscle progenitors cells	^[^ ^124^ ^]^
Decellularized muscle ECM	Hydrogel	Rat (*n* = 9–11)	Excision of femoral artery	Increased	Increased arteriole and capillary density	Improved muscle remodeling: Increased in Pax7+ satellite cells and CNFs	^[^ ^121a^ ^]^
Fibrin	Sealant	Rabbit (*n* = 6)	Excision of femoral artery	Increased	Increased capillaries area		^[^ ^125^ ^]^
Fibrin	Microspheres	Rabbit (*n* = 6)	Excision of femoral artery	Increased collaterals	Increased capillary density		^[^ ^126^ ^]^

Fibrin has been used with different engineered architectures showing an ability to induce revascularization and reperfusion when intramuscularly injected in a rabbit model of CLTI.^[^ ^125^, ^126^ ^]^ In particular, a fibrin sealant was found to induce the formation of collateral vessels and capillaries.^[^ ^125^ ^]^ Furthermore, fibrin particles prepared by treating fibrinogen from bovine plasma with thrombin, were able to cause and increase in capillary density and recovery of reperfusion in the ischemic legs.^[^ ^126^ ^]^ Additionally, hybrid composites like a fibrin‐chitosan hydrogel was designed by the use of PEG solution, chitosan, fibrinogen and thrombin solution. The most suitable ratio of each component was optimized by encapsulating cells in vitro. The combined formulation improved the blood reperfusion and the capillary formation but also rescued the limb from necrosis and amputation in a mouse model.^[^ ^123^ ^]^ Recently, heparan sulfate was solely administered since it has been noticed its key role in the binding of VEGF to receptors on endothelial cells to initiate the angiogenesis. The use of this GAG was found to improved blood reperfusion in a CLTI mouse model.^[^ ^122^ ^]^

While fibrin or heparan sulfate was able to influence on the vascularization, ECM‐mimic hydrogel, have additionally shown the ability to induce muscle remodeling.^[^ ^106^ ^]^ Indeed, an injectable hydrogel obtained from porcine skeletal muscle decellularized ECM showed an increase not only in vascular cells but also in regenerating muscle fibers and progenitor cells in a rat model of hindlimb ischemia.^[^ ^124^ ^]^ This decellularized ECM hydrogel was also compared with human umbilical cord ECM hydrogel and showed a superior ability to improve fiber morphology and to activate paired box protein 7 (Pax7)+ satellite cells into the muscle regeneration program.^[^ ^121a^ ^]^ To engineer ECM like materials with the potential of triggering pro regenerative phenotypes of cells in ischemic tissues is an effective way of promoting self‐recovery of both capillaries and the skeletal muscle.

## 4 Concluding Remarks and Future Directions

Recently, biomaterials have emerged as undisputed protagonists in tissue engineering approaches that aim to induce revascularization and regeneration for CLTI applications. Increasing number of preclinical trials have proved the potential of biomaterials to overcome the limitations highlighted in clinical trials in growth factors, gene and cell therapy, improving their efficacy. Furthermore, biomaterials alone have shown significant angiogenic and regenerative potential in preclinical CLTI models. From a manufacturing perspective, this approach represents the most economical way to translate on a large scale. Nevertheless, extensive research encouraged the use of biomaterials, but difficulties must still need to be overcome before implementation into the clinic. Biomaterials also offered tunability to incorporate additional factors such as cells or growth factors. However, to include cells lead to a reduction in shelf life and substantially increases the cost of a potential scale‐up. On the other hand, protein addition presents similar drawbacks. The synthesis of engineered analogues of the growth factors or cytokines by new manufacturing methods is a new strategy with high translational potential.

To date, most of the studies are mainly focused on the effects of the biomaterial‐based strategies on the revascularization aspect. Albeit the effect on muscle regeneration, fibrosis and inflammation remain poorly explored. Because intact and functional muscle is necessary for the efficacy of revascularization therapy, approaches to induce muscle regeneration are required.

Furthermore, the safety and the potential cytotoxicity, especially of nucleic acid and cells‐related products, need long‐term investigation. The use of ECM‐based materials to restore GAG equilibrium in damaged ischemic tissue without any inherent additional risk seems promising. The ability of these materials to provide a structure that offers mechanical and biochemical clues could overcome the main limitations of the existing therapies used in the clinic. GAG based formulations are bioactive actors that trigger activation of pro‐regenerative and pro‐angiogenic pathways that lead to the formation of new vessels. These results support and encourage the continued engineering of biomaterials as therapeutics when targeting ischemic diseases such as CLTI. From a scale‐up perspective, a biomaterial‐alone approach is the most suitable, as to include additional therapeutics considerably increases the cost and manufacturing time. However, from a therapeutic point of view, the body of research supports the needed of a combinatorial approach to obtain an effective therapy for a highly complex clinical need as CLTI. Therapies using ECM materials combined with other active players such as stem cells, exosomes, and a sustained release of protective factors, such as NO, are likely to overcome the limitations of each component.

Additionally, there is an urgent need for in‐depth studies of the mechanisms of action of these biomaterial systems. Recent advances in high throughput analysis such as genomics, proteomics and glycomics can be used to understand the molecular basis of the pro‐angiogenic action of the biomaterial constructs. Validating the safety and understanding of the molecular mechanism will only facilitate the clinical experimentation phase.

## Conflict of Interest

The authors declare no conflict of interest.
